# Molecular Characterization Of Pathogenic *Salmonella Spp* From Raw Beef In Karachi, Pakistan

**DOI:** 10.3390/antibiotics9020073

**Published:** 2020-02-10

**Authors:** Muhammad Altaf Hussain, Wan Wang, Changbao Sun, Liya Gu, Zhijing Liu, Tong Yu, Yasin Ahmad, Zhanmei Jiang, Juncai Hou

**Affiliations:** 1 Key Laboratory of Dairy Science, College of Food Science, Northeast Agricultural University, Harbin 150030, China; draltafdawar@gmail.com (M.A.H.); 13159806631@163.com (W.W.); sunchangbao@126.com (C.S.); Andrea_hh@163.com (L.G.); liuzhijing22@126.com (Z.L.); 15546092358@163.com (T.Y.); jiangzhanmei@163.com (Z.J.); 2 Department of Microbiology, Quaid-i-Azam University, Islamabad 45710, Pakistan; ahmadyasin9312@gmail.com

**Keywords:** *Salmonella*, raw beef, public health, antibiotic resistance, virulence factors

## Abstract

The aim of the present study was to estimate the prevalence of *Salmonella* and investigate the dominant serovars distribution in raw beef and to screen the isolated serovars for the prescense of beta-lactamases and virulence genes. A total of 150 samples of raw beef sold at butcher shops (*n* = 75) and supermarkets (*n* = 75) in Karachi city were collected (50 samples each from muscles, lymph nodes, and minced beef). The samples were cultured according to the ISO-6579-1guidlines. The overall prevalence of *Salmonella* strains was found to be 21.34%. A total of 56 isolates of *Salmonella* belonging to four serogroups (*Salmonella* Pullorum, *Salmonella* Enteritidis, *Salmonella* Typhimurium and *Salmonella* Choleraesuis) were isolated from beef muscles (12%), lymph nodes (24%) and minced beef (28%) samples collected from butcher shops (av. 21.34%). No *Salmonella* was detected in beef samples collected from supermarkets. *S*. Enteritidis contamination was highest (37.5%), followed by *S*. Choleraesuis (30.4%), *S*. Pullorum (19.6%) and *S*. Typhimurium (12.5 %). Antibiotic susceptibility testing revealed that *Salmonella* isolates were highly resistant to Oxytetracycline (90%), Ampicillin (90.5%), Amoxicillin (81.1%), Tetracycline (76%), Neomycin, (79.8%) and Ciprofloxacin (61.4%). The *Salmonella* isolates examined were more susceptible to the Cephalosporin antibiotics such as Cefixime (43.2%), Cefepime (48.2) and Cefoxitin (49.8%). PCR based screening of bla*TEM*, bla*CTX-M* and bla*SHV* revealed that bla*CTX-M* and bla*TEM* were the dominant resistant genes in *S*. Enteritidis and *S*. Typhimurium followed by *S*. Pullorum and *S*. Choleraesuis whereas bla*SHV* was the least detected beta-lactamase in *Salmonella* isolates. Virulence genes screening revealed that at least five genes were present in all the serovars, highest being present in *S*. Enteritidis (12/17) and *S*. Typhimurium (12/17). *S*. Cholerasuis (5/17) carried the least number of virulence genes followed by *S*. Pullorum (6/17). The present data suggest that beef samples from butcher shops of Karachi city are heavily contaminated with MDR *Salmonella*. The presence of resistance and virulence genes in MDR strains of *Salmonella* may play a significant role in transmission and development of *Salmonella* infection in humans.

## 1. Introduction

Foodborne diseases pose a significant threat to global health. Non-typhoidal *Salmonella* serovars are a major cause of food-borne illnesses both in developed and developing countries of the world [1]. According to recent epidemiological data, 94 million cases of salmonellosis are reported globally, of which 83.3 million cases are due to the consumption of contaminated food [2]. In USA, 40,000 cases of non-typhoidal salmonellosis are reported annually [3]. Similarly, the prevalence rate of salmonellosis in European Union was 23.4 cases per 100,000 population in 2014 [4]. The prevalence rate of salmonellosis and its underlying causes may vary significantly with geographical boundaries. Foods of animal origin have been considered a significant cause of salmonellosis in humans. The infection is usually acquired through the consumption of contaminated food such as poultry, mutton, pork, beef and their products [5,6]. In the USA, salmonellosis is caused by the consumption of contaminated eggs, poultry and beef, and particularly minced beef [7].

Meat, either in traditional cooked form or in its processed forms, is considered as a tremendous source of proteins and vitamins for humans. Meat is rich in high quality animal proteins, vitamin B complex, and most of the minerals essentially required for nutritionally balanced food [8]. Among meats, beef is ranked 3rd in consumption globally. It is estimated that 25 percent meat production consist of raw beef, after pork and poultry that constitute 38 and 30 percent of the total meat production in the world, respectively. Brazil is the biggest beef exporter of the world (1.850 million tons), followed by India, Australia, USA and New Zealand with 1.850, 1.385, 1.120 and 0.580 million tons of beef exports, respectively. The USA is the largest beef consumer in the world, followed by Brazil, China and the European Union [9]. Pakistan produces 4478 thousand tons of meat, and annual beef production is 2227 thousand tons, while Pakistan exported 48.8 thousand tons of meat during 2018 [10].

The contamination of beef with *Salmonella* could occur at various stages such as slaughtering, skinning, evisceration, transportation and cutting in retail markets which can pose a significant threat to public health. Certain serotypes are associated explicitly with cattle; which mainly include *S*. Dublin, *S.* Typhimurium, *S.* Kentucky, *S.* Hadar and *S*. Enteritidis [11,12]. *Salmonella* has been widely reported in cattle all over the world [13,14,15], and infected animals may shed organisms in their feces without showing any clinical signs of the disease. Therefore, the cattle may carry these undetected organisms into abattoir at the time of slaughtering [16].

The extensive use of antibiotics for prophylaxis and growth promotion in cattle and other livestock animals have resulted in the emergence of MDR strains of *Salmonella* [17,18]. There are various research reports describing the isolation of MDR (resistant to three or more classes of antimicrobials) *Salmonella* strains displaying resistance to clinically important antimicrobials such as third-generation cephalosporins, fluoroquinolones and carbapenems [19,20,21,22]. Gastrointestinal salmonellosis usually doesn’t require antibiotic treatment however; invasive salmonellosis requires immediate treatment with appropriate antibiotics [23].

Foodborne diseases are responsible for causing significant economic and health problems in Pakistan [24]. The rate of *Salmonella* prevalence at the public level is about 0.45% (451.7 cases per 100,000 population). The high rate of prevalence and antibiotic resistance of *Salmonella* in poultry meat hasbeen reported in several cities of Pakistan [25,26]. Due to the lack of proper surveillance systems, the data regarding *Salmonella* prevalence in cattle population and raw beef is very rare. Moreover, the antimicrobial resistance and virulence profile of *Salmonella* serovars in cattle population and raw beef were never investigated in Pakistan. Hence, for the first time, the current study was performed to examine the prevalence, antibiotic resistance pattern and virulence profile of *Salmonella* in raw beef samples collected from butcher shops and supermarkets of Karachi city.

## 2. Results

### 2.1. Detection of Salmonella in Raw Beef Samples from Different Body Parts

**Figure 1:**
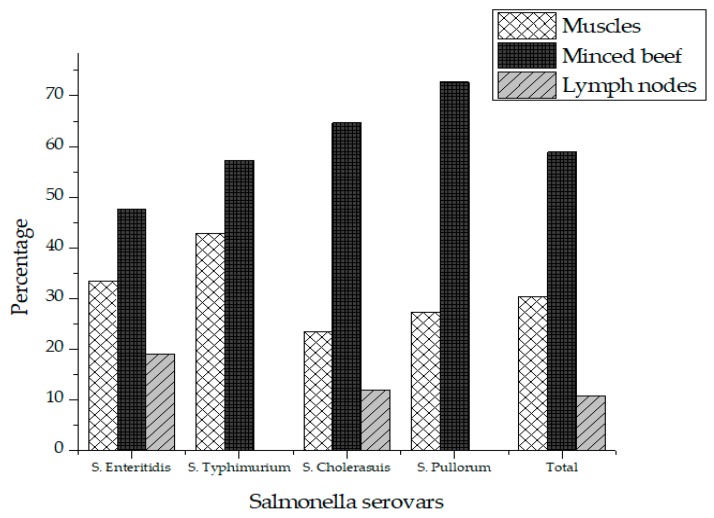
Prevalence of *Salmonella* in total positive samples.

Out of 75 different beef samples from butcher shops, 16 (21.3%) tested positive for *Salmonella*, while no sample was tested positive for *Salmonella* collected from supermarkets in Karachi city. A two tailed *p*-value was obtained using student’s T-test for comparison of *Salmonella* prevalence in butcher shops and supermarkets. A two-tailed *p*-value of 0.0001 was obtained, which by conventional criteria, is considered extremely statistically significant. The contamination rate of *Salmonella* was 12% (*n* = 3), 28% (*n* = 7) and 24% (*n* = 6) in the muscles, minced beef and lymph node respectively (Table 1). A total of 56 different isolates of *Salmonella* were recovered from the positive samples comprising of four different serovars in which *S*. Enteritidis (37.5%) was the most prevalent serovar followed by *S*. Cholerasuis (30.4) and S. Pullorum (19.6%). *S*. Typhimurium (12.5%) was the least detected serovar in raw beef as shown in Table 2. *Salmonella* was most prevalent in minced meat (58.9%) followed by muscles (30.4%) and lymph nodes (10.7%) as shown in Table 2. Figure 1 represents the prevalence of *Salmonella* serovars in muscles, lymph nodes and minced beef.

### 2.2. PCR Screening of Virulence and Resistance Genes in Salmonella Isolates

**Figure 2:**
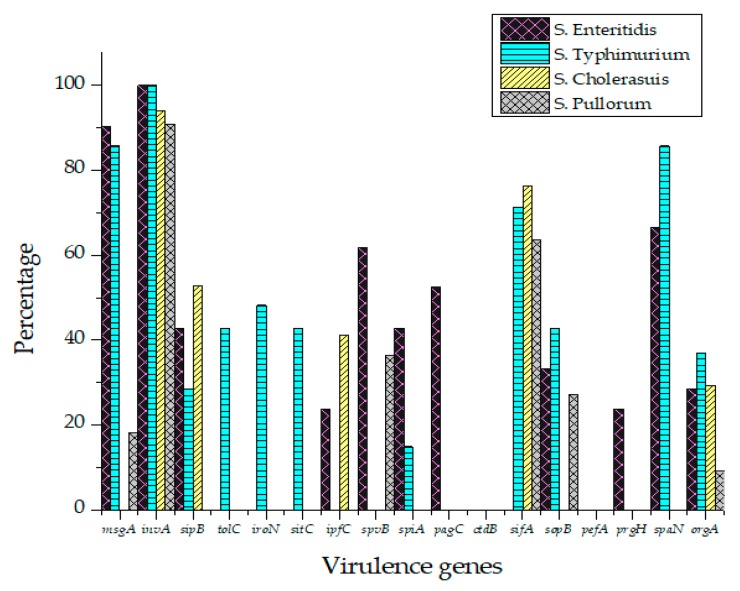
Prevalence of virulence factors in *S*. Enteritidis, *S*. Typhimurium, *S*. Pullorum and *S*. Choleraesuis.

**Figure 3:**
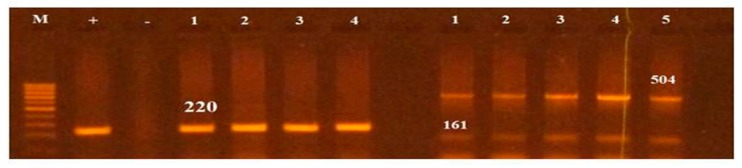
PCR based screening of *sopB* (220), *tolC* (161bp) and *spaN* (504bp). Lane M represents 100bp ladder.

**Figure 4:**
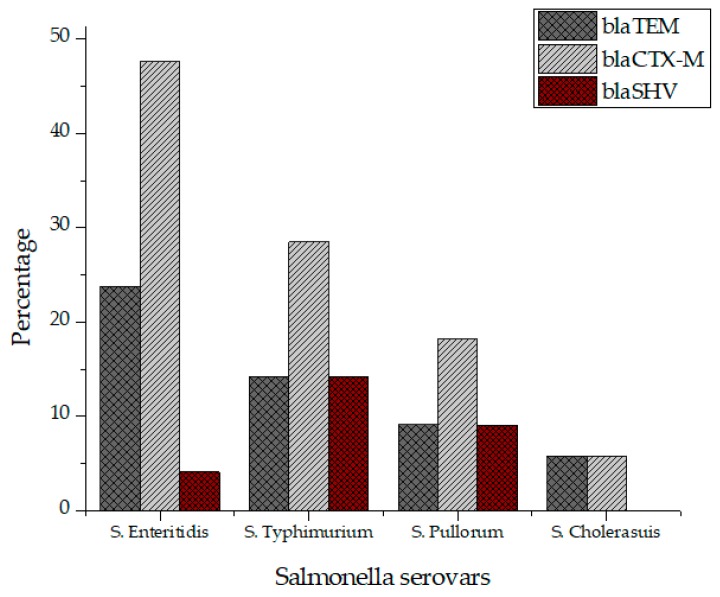
Prevalence of beta lactamases in *S*. Enteritidis, *S*. Typhimurium, *S*. Pullorum and *S*. Choleraesuis.

All *Salmonella* isolates were screened for seventeen virulence factors through conventional PCR. The data showed in Figure 2 represent the relative frequencies of virulence genes in *Salmonella* serovars. Virulence gene *invA* was found in all four groups of serovars while *sipB, sitC, spvB* and *spiA* were detected in at least two different serovars. Virulence factors *msgA, ipfC, spaN, orgA, sopB* and *sifA* were detected in at least three different serotypes. *S*. Enteritidis and *S*. Typhimurium each harbored most virulence genes (12/17) followed by *S*. Pullorum (6/17) and *S*. Cholerasuis (5/17). Figure 3 represents the gel electrophoresis result of amplified product of *sopB*, *tolC* and *spaN* genes. Among beta-lactamases, bla*TEM* was most prevalent in *S*. Enteritidis isolates followed by *S*. Typhimurium (14.5%) and *S*. Pullorum (2%). Prevalence of bla*CTX-M* was highest in *S*. Enteritidis isolates (49.6%) succeeded by *S*. Typhimurium (28%), *S*. Pullorum (11%) and *S*. Choleraesuis (1.8%). bla*SHV* was the least detected beta-lactamase having prevalence of 2.6% in *S*. Enteritidis, 5% in *S*. Typhimurium and 2% in *S*. Pullorum. bla*SHV* was not detected in *S*. Choleraesuis. Figure 4 represents prevalence of beta-lactamases in *Salmonella* isolates.

### 2.3. Antimicrobial Resistance in Salmonella Isolates

**Figure 5:**
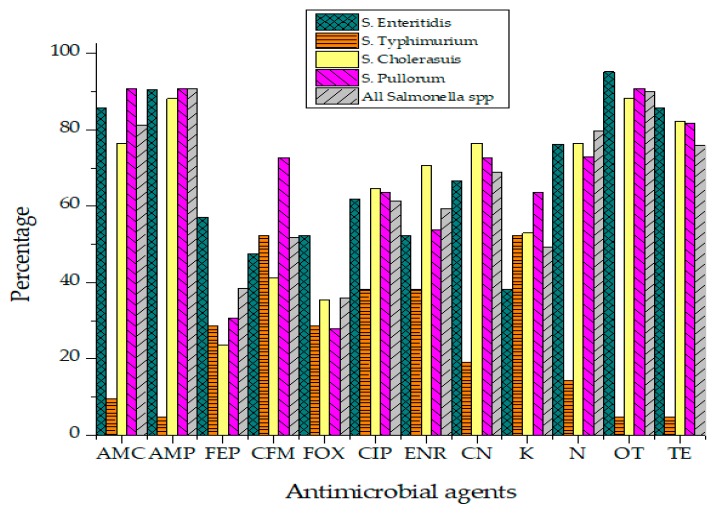
Resistance percentage of *Salmonella* to various antibiotics.

Antibiotic susceptibility results of *Salmonella* isolates against 12 different antibiotics have been presented in Figure 5. *Salmonella* isolates were categorized into "Resistant," "Intermediate" and "Sensitive" based upon the instruction of the Clinical and Laboratory Standards Institute (CLSI, 2019).

Not a single isolate of *Salmonella* was susceptible to all tested antibiotics. The highest resistance was recorded against Ampicillin (90.9%), Oxytetracycline (90%), Amoxicillin (81.1%), Neomycin (79.8%) and Ciprofloxacin (61.4%) as represented in Table 3. Isolates were found susceptible to Cephalosporins such as Cefoxitin (49.8%), Cefepime (48.2%) and Cefixime (43.2%). Enrofloxacin (32.9%) also showed activity against *Salmonella* isolates of raw beef origin. *Salmonella* isolates phenotypically resistant to three or more than three classes of antibiotics were classified as MDR strains. MDR phenotype was observed in 33.9% *Salmonella* isolates. While 23.2% of isolates were resistant to 4–5 different antibiotics. Isolates showing resistance against more than seven different antibiotics were found to be 12.5% of all *Salmonella* isolates. All *Salmonella* Typhimurium isolates were resistant to Ampicillin while none was found sensitive to Ciprofloxacin (R = 78.4%, I = 28.6%). No isolate of *S*. Pullorum was susceptible to Gentamicin, Ampicillin, Amoxicillin and Ciprofloxacin. Statistically, the association of antibiotic resistance in specific serovars and meat sources such as muscles, lymph nodes and minced beef was not significant. Isolates exhibited antibiotic resistance irrespective of their source of isolation.

### 2.4. Morphological and Biochemical Characteristics

**Figure 6:**
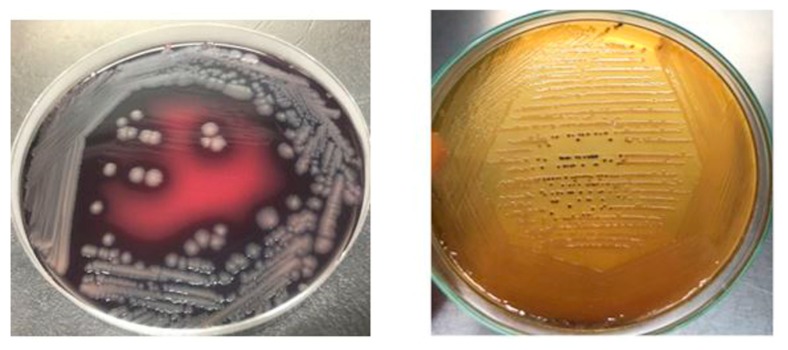
*Salmonella* culture morphology on SS agar and XLD agar.

*Salmonella* isolates recovered from raw beef samples were morphologically characterized through gram staining. *Salmonella* isolates grown on XLD agar resulted in red colonies with black centers while on SS agar *Salmonella* resulted in transparent or straw-colored colonies with black centers as depicted in Figure 6. *Salmonella* Enteritidis stained pink with Gram-staining, actively motile, ranged in its size from 0.3 to 1.6mm in diameter and occurred in singles/short chains. *S*. Typhimurium and *S*. Cholerasuis displayed morphological characteristics similar to *S*. Enteritidis but produced less black colonies on XLD and SS agar. 

## 3. Discussion

*Salmonella,* an anthroponotic pathogen, is the second leading cause of food-borne illnesses all over the world. Food of animal origin such as beef meat is deemed to be the vehicle of transmission of *Salmonella* to humans [27,28]. The aim of this study was to determine *Salmonella* contamination in raw beef sold at wet markets and supermarkets. To the best of our knowledge, this is the first report of prevalence and characterization of *Salmonella* isolates detected in raw beef in Karachi city, Pakistan. The *Salmonella* prevalence in raw beef samples was 21.34% which is in agreement with other research findings [29,30,31,32]. The high level of *Salmonella* contamination in various forms of raw beef such as muscles, minced beef and lymph nodes indicates poor hygienic conditions and practices adopted by workers at various stages of beef processing. Moreover, the refrigeration conditions for beef are almost nonexistent at butcher shops which can significantly accelerate the growth of *Salmonella* and other foodborne pathogens present in beef. It is worth to note that not a single raw beef sample was positive for *Salmonella.* Due to the lack of adequate surveillance data regarding food items in Karachi city, it is difficult to compare the high contamination rate in retail beef shops with zero contamination in supermarkets’ raw beef. However, supermarkets do have good hygienic practices while handling and cutting raw beef as compared to retail beef markets in Karachi city. The prevalence of *Salmonella* in beef samples in our research study is higher as compared to previous research studies [33,34,35,36]. However, some research studies have reported more than 60% prevalence in beef samples [37,38,39,40]. The *Salmonella* prevalence in beef is much lower in developed countries like USA due to strict control measures. The prevalence of *Salmonella* in minced meat has been reported up to 12 percent by [41] which indicates high microbiological risk because *Salmonella* must not be present in meat when it is sold. Similar results have also been reported by [42]. Some research studies have reported 5%–20% prevalence of *Salmonella* in meat [43,44]. According to our findings, *S*. Enteritidis, *S*. Cholerasuis, *S*. Typhimurium and *S*. Pullorum were the dominant serovars found in raw beef. The prevalence of *Salmonella* serovars in raw beef or meats such as poultry and pork can vary considerably among different regions of the world. The difference could be attributed to changes in temperature, rainfall and humidity. *Salmonella* Enteritidis was the most prevalent serovar with prevalence rate of 37.5 which is supported by the findings of Sallam et al. Although *S*. Pullorum is routinely found in poultry meat being potential pathogens of poultry industry, however, it can be also be detected in raw beef due to cross contamination with poultry meat. There are few reports describing the prevalence of *S*. Pullorum in beef and other meats i.e., fish, pork etc. [42].

*Salmonella* induced foodborne diseases have gained more significant attention because of food poisoning causality. This study indicates that beef samples from butchers’ shops of Karachi city are heavily contaminated *S*. Enteritidis (37.5%) and *S*. Cholerasuis (31.25%). This level of contamination in beef suggests poor sanitary conditions of raw meat production where it is being produced. This also indicates fecal pollution close to the butcher shops or where they slaughter the animals that start at direct contact of worker’s hands with knives for skinning [45]. Such contamination may also find access while visceral organs are washed and from water while the meat is rinsed and washed. In this study prevalence of *S*. Enteritidis and *S*. Typhimurium in beef could be considered as distinct population suggests that meat animals may be the predominant source of contamination [46,47].

*Salmonella* isolates of raw beef origin exhibited high resistance to antibiotics of therapeutic importance such as Tetracycline, Oxytetracycline, Ampicillin, Amoxicillin and Ciprofloxacin which reflects their high use in the livestock industry in Pakistan [39]. The overuse and misuse of antibiotics in livestock industry have greatly contributed to the spread of MDR bacteria especially *Salmonella* to humans via the food chain [48]. The high level of resistance exhibited by *Salmonella* isolates to Amoxicillin, Ciprofloxacin and Tetracycline could pose serious threats to human health because they are the drug of choice in cases of invasive salmonellosis [49].

Virulence of a bacterial species can be combined attributed to both resistance and virulence genes. *Salmonella* serovars can carry a multitude of different resistance and virulence genes. Data regarding virulence characterization of *Salmonella* isolates from raw beef origin is rare. However, researchers have well-characterized *Salmonella* isolates from pork and poultry origin [20,50]. In our research study, we screened *Salmonella* isolates for seventeen (17) well-known virulence factors. For the first time in Karachi, Pakistan, we reported the prevalence of virulence genes in *Salmonella* isolates from beef origin. According to our PCR results, *S*. Enteritidis and *S*. Typhimurium carried a wide range of Virulence determinants genes as compared to *S*. Pullorum and *S*. Cholerasuis. All strains of *S*. Enteritidis were positive for *invA* gene which is a widely used gene for the genus level identification of *Salmonella* [51,52,53]. *SopB* is a Type-III secreted effector proteins, which helps in the invasion and maintenance of *Salmonella* containing vacuole (SVC), while *sipA* facilitates entry into host cells [54]. The prevalence of *sopB* in *S*. Enteritidis, *S*. Typhimurium and *S*. Pullorum was found to be 35%, 38% and 27.2% isolates respectively which indicate their zoonotic potential. A previous study on virulotyping of *S*. Enteritidis and *S*. Typhimurium isolates from poultry meat in Faisalabad found *sopB* in 81.8% and 91.1% isolates of *S*. Enteritidis and *S*. Typhimurium respectively [55]. The *Salmonella* isolates were screened for beta-lactamases i.e., *blaSHV*, *blaTEM* and *blaCTX*-M. *S*. Enteritidis and *S*. Typhimurium carried highest percentage of *blaTEM* and *blaCTX*-M. The carriage of beta-lactamases by *Salmonella* isolates reflects the fact that bacteria carry antibiotic resistance genes for enhanced survivability in an environment with selective pressure in the form of antimicrobials [56]. It is a matter of fact that antimicrobials are extensively used in cattle and other livestock animals for prophylaxis and growth promotion, which ultimately resulted in the emergence of multi and extensively drug-resistant strains of *Salmonella* [39].

## 4. Materials and Methods

### 4.1. Samples Collection and Processing

The study was carried out on samples of raw beef sold at butcher shops and supermarkets in Karachi, Pakistan.. Meat samples from muscles (*n* = 25), lymph nodes (*n* = 25) and minced beef (*n* = 25) were collected from butcher shops (*n* = 75) and supermarkets (*n* = 75) [Total *n* = 150]. These samples were aseptically collected in sterile plastic bags that were kept in an icebox and brought to the Central Veterinary Diagnostic Laboratory Tandojam within 4–6 hours under cold conditions for further processing.

### 4.2. Isolation of Salmonella

For isolation of *Salmonella*, about 25 g of the sample was aseptically added to Buffered Peptone Water (BPW) (Himedia, India) and incubated at 37 °C for 24 hours. The next day, 100 μL sample from BPW (Oxoid, UK) was added to 10mL of Tetrathionate broth (Himedia, India) containing Potassium Iodide and Iodine solution as recommended by the manufacturers. The tubes were incubated at 37 °C for 24 hours. The day after incubation, 100 μL of liquid sample from Tetrathionate broth was streaked over the surface of the Salmonella-Shigella (SS) agar and Xylose Lysine Deoxycholate (XLD) agar and incubated 37 °C for 24 hours. The plates were observed for positive growth the day after incubation.

### 4.3. Identification of Salmonella

The colonies with black centers on SS and XLD agar were assumed to be *Salmonella*. The suspected *Salmonella* colonies were initially subjected to traditional morphological and biochemical testing such as Gram staining, catalase test, Triple Sugar Iron test, Citrate utilization test and MR-VP test.

The confirmed diagnosis consisted of a laser desorption (matrix-assisted) profile. Briefly, around 10 mg cells of the isolate were suspended in 300 μL sterile water. A 900 μL absolute ethanol was added and mixture was centrifuged for 2 min at 10,000 rpm. The supernatant was discarded and pellet was suspended in 50 µL 70% v/v formic acid. After addition of 50 µL acetonitrile, blend was centrifuged for 2 min at 10,000 rpm and 1 μL clear supernatant was shifted to MALDI target for drying, and then 1 µL alpha-Cyano-4-hydroxycinnamic acid was added to mixture. Before running MALDI, bacterial strain Nussle was analyzed to work as the standard for the calibration and positive control. Mass spectrometry analysis (MALDI-TOF) was done by mass spectrometer (GmbH, Germany), that identified the spectra automatically through the software fabricated by GmbH, Germany named as Bruker Bio Typer-1.1. Kauffman white type scheme serotype was tested with side agglutination having different O and H antisera.

### 4.4. Antibiotic Susceptibility Profiling

*Salmonella* isolates were assayed for their antibiotic susceptibility to Gentamycin (10 μ), Neomycin (30 μ), Kanamycin (30 μ), Cefixime (05 μ), Cefoxitin (30 μ), Cefepime (30 μ), Ciprofloxacin (05 μ), Enrofloxacin (05 μ), Tetracycline (30 μ), Oxytetracycline (30 μ), Ampicillin (10 μ) and Amoxicillin (30 μ) by disk diffusion method according to CLSI, 2019 guidelines. Inhibition zones were measured to determine the sensitivity or resistance of the isolates to the antibiotics. The reason behind choosing the above antimicrobials was that these are commonly used for treatment of people suffering from *Salmonella* infection. The disk diffusion method was used to check the susceptibility or resistance of isolates to various antimicrobials. Cut-off point guidelines from the CLSI M100 chart 2A were used to categorize strains into “Resistant”, “Intermediate” or “Susceptible”. Few colonies of the test organism with the same morphological characteristics were picked up with the help of a sterile cotton swab and mixed with nutrient broth. The turbidity of the inoculum suspension was compared with 0.5 McFarland standard solution. The bacterial suspension was spread across the entire surface of MHA plate with the help of sterile cotton swab. The MHA plates were allowed to dry for a few minutes. The individual antibiotic discs were then placed and pressed slightly on the MHA plate with an equal distance. The plates were then placed in an incubator set at 37 °C for 24 hours. Inhibition zones were observed after 24 hours of incubation and measured to the degree of area from the disk center. Isolates exhibiting resistance against three or more different classes of antibiotics were considered as multidrug-resistant (MDR) strains.

### 4.5. DNA Extraction and Purification

DNA was extracted using heat boiling method. Briefly, few well isolated *Salmonella* colonies were suspended in 1 mL Brain Heart Infusion Broth (Oxoid, UK) and incubated overnight at 37 °C. The next day, the Eppendorf tubes were centrifuged at 14,000× *g* for 10 min. The pellet was suspended in freshly prepared TE buffer (10mM Tris-HCl, 1mM EDTA) and spun for a short time to resuspend the pellet. The Eppendorf tubes were kept in water bath set at 100 °C for 5 min and then immediately shifted to −80 deep freezer for 3 min. This freeze-thaw cycle was repeated three times to lyse the cells completely. The tubes were centrifuged at 14,000× *g* for 5 min at 4 °C temperature. The supernatant was saved in another clean tube for further use in molecular characterization. The concentration and purity of DNA were checked through Nanodrop.

### 4.6. PCR Screening of Resistance and Virulence Genes

Various resistance genes conferring resistance to commonly used drugs of choice in humans were targeted through PCR. Commonly reported antibiotic resistance genes from various classes of antibiotics were selected which included *β*-lactamases *TEM*, *SHV*, and *CTX-M*. Different virulence factors were screened in *Salmonella* isolates through PCR which include *spaN, msgA, sipA*, *invA*, *tolC, iroN, sitC, lpfC, spvB, spiA, pagC, cdtB, sifA, sopB, pefA, prgH,* and *orgA*. The primer sequences used for amplification of target genes are summarized in Table 4. The PCR amplification was carried out in 25 μL reaction PCR tubes containing 12.5 μL mater mix (amaR OnePCR^TM^), 1 μL of forward and reverse primer, 5 μL DNA template and 3 μL molecular grade PCR water. The thermal cycler (Master Cycler; Eppendorf, Germany) conditions used in this study for amplification of target genes were the same except for annealing temperatures. The reaction conditions were as following; initial denaturation at 95 °C for 5 min followed by 35 cycles of denaturation at 95 °C for 1min, primer annealing for 45 s, and final extension at 72 °C for 5 min. The PCR product was electrophoresed on 1.5% agarose along with 100 bp DNA ladder (Genedirex). The gel was visualized using gel documentation system (BioSens SC 645).

### 4.7. Statistical Analysis

The data are presented as mean ± SEM. Computer software SPSS version 20.0 was used for statistical analysis and variation between the groups was determined through the Student’s t-test. Variations were considered statistically significant at * *p* < 0.05.

## 5. Conclusions

This study concludes that raw beef sold at butcher’s shops in Karachi city are heavily contaminated with *Salmonella* species. Despite the fact that beef is usually not consumed in raw form, it can still pose serious threats to public health because of possible cross-contamination to other foods and the consumption of improperly cooked meat. This study indicated that raw beef may be a vehicle of MDR serovars of *Salmonella*. The recovered isolates exhibited phenotypic resistance to many antimicrobials of therapeutic importance. Moreover, the isolates were positive for important virulence factors which can play role in pathogenesis if consumed in food by humans.

## Figures and Tables

**Table 1:** Prevalence of *Salmonella* spp. in raw beef meat samples sold at butcher shops and supermarkets.

Source	Sample Type							
	Muscles		Minced Beef		Lymph Nodes		Total Positive/Total Samples	%
	Positive/Total Samples	%	Positive/Total Samples	%	Positive/Total Samples	%		
Butcher Shops	3/25	12	7/25	28	6/25	24	16/75	21.3
Supermarkets	0/25	Nil	0/25	Nil	0/25	Nil	0/75	Nil

**Table 2:** Prevalence of *S*. Enteritidis, *S*. Typhimurium, *S*. Cholerasuis and *S*. Pullorum in different beef samples.

Source									
Meat Type	Butcher Shops				Supermarkets				Total Strains in Each Sample (%)
	*S*. Enteritidis	*S*. Typhimurium	*S*. Cholerasuis	*S*. Pullorum	*S*. Enteritidis	*S*. Typhimurium	*S*. Cholerasuis	*S*. Pullorum	
Muscles	7 (33.3)	3 (42.8)	4 (23.5)	3 (27.3)	0	0	0	0	30.4
Minced Meat	10 (47.6)	4 (57.2)	11 (64.7)	8 (72.7)	0	0	0	0	58.9
Lymph Nodes	4 (19.04)	0	2 (11.8)	0	0	0	0	0	10.7
Total Serovars (%)	37.5	12.5	30.4	19.6					

**Table 3:** Antimicrobial resistance profile of *Salmonella* spp.

Antibacterial Agent	*Salmonella* isolates														
	*S*. Enteritidis			*S*. Typhimurium			*S*. Cholerasuis			*S*. Pullorum			All *Salmonella* spp		
	R%	I%	S%	R%	I%	S%	R%	I%	S%	R%	I%	S%	R%	I%	S%
Amoxicillin	85.7	4.8	9.6	71.4	-	28.6	76.5	-	23.5	90.9	-	9.09	81.1	1.2	17.7
Ampicillin	90.6	4.7	4.7	100	-	-	88.2	-	17.6	90.9	-	9.09	90.9	1.2	7.8
Cefipime	57.1	14.2	28.7	42.7	-	57.1	23.5	11.76	64.7	30.7	27.2	42.1	38.5	13.3	48.2
Cefixime	47.6	-	52.4	42.8	-	57.2	41.2	11.7	47.03	72.7	9.09	18.2	51.7	5.2	43.2
Cefoxitin	52.4	19.04	28.6	28.5	14.2	57.3	35.3	23.5	41.2	27.8	-	72.2	36	14.2	49.8
Ciprofloxacin	61.9	-	38.1	71.4	28.6	-	64.7	5.88	29.4	63.6	36.4	-	61.4	10.7	27.7
Enrofloxacin	52.4	9.5	38.1	57.1	14.2	28.7	70.6	-	29.4	53.8	17.6	28.6	59.4	7.7	32.9
Gentamicin	66.6	14.4	19.04	71.4	-	28.6	76.5	-	23.5	72.7	36.4	-	68.8	10.7	20.7
Kanamycin	38.09	9.5	52.4	42.8	57.1	14.3	52.9	17.6	35.3	63.6	27.3	9.09	49.3	25.9	24.7
Neomycin	76.2	9.52	14.3	85.7	-	14.3	76.5	-	23.5	72.8	-	18.2	79.8	2.4	17.6
Oxytetracycline	95.2	-	4.8	85.7	14.3	-	88.2	-	11.8	90.9	-	9.09	90	3.5	7.6
Tetracycline	85.7	9.5	4.8	71.4	-	28.6	82.3	11.8	23.5	81.8	-	18.2	76	5.3	18.7
No of isolates resistant to multiple classes of antibiotics															
0–1	7			2			5			3			17		
2–3	5			3			7			4			19		
4–5	6			2			3			2			13		
5>	3			0			2			2			7		

**Table 4:** Primer’s oligonucleotide sequences.

Genes	Oligonucleotide Sequences of Primers	Product Size	Annealing Temperature	References
*TEM*	5′-TTCGCCTGTGTATTATCTCCCTG-3′ 5′-TTAGCGTTGCCAGTGYTCG-3′	854	54 °C	[56]
*CTX-M*	5′-ATGTGCAGYACCAGTAARGTKATGGC-3′ 5′-TGGGTRAARTARGTSACCAGAAYCAGCGG-3′	593	62 °C	[57]
*SHV*	5′-GGGTTATTCTTATTTGTCGC -3′ 5′-TTAGCGTTGCCAGTGCTC -3′	615	60 °C	[58]
*msgA*	5′-GCCAGGCGCACCCGAAATCATCC-3′ 5′-GCGACCAGCCAGATATCAGCCTCTTCAAAC-3′	190	65 °C	[59]
*invA*	5′-CTGGCGGTGGCTTTTGTTGTCTTCTCTATT-3′ 5′-AGTTTCTCGCCCTCTTCATGCGTTACCC-3′	1070	65 °C	[59]
*sipB*	5′-GGACGCCGCCGGGGAAAAACTCTC-3′ 5′-ACACTCCCGTCGGCGCCTTCACAA-3′	875	56 °C	[59]
*tolC*	5′-TACCCAGGCGCCAAAAGAGGCTATC-3′ 5′-CCCGCGGTTATCCAGGGCTTGTTGC-3′	161	56 °C	[59]
*iroN*	5′-ACTGGCACGGCTCGGTGTCGCTCTAT-3′ 5′-CGCTTTACCGCCCTTCTGCCACTGC-3′	1205	56 °C	[59]
*sitC*	5′-CAGTATATGCTCAAGGCGATGTGGGTCTCC-3′ 5′-CGGGGCGAAAATAAGGGCTGTGATGAAC-3′	768	55 °C	[59]
*lpfC*	5′-GCCCCGCCTGAAGCGTGTGTTGC-3′ 5′-AGGTCGCCGCTGTTGGAGGTTGGATA-3′	641	60 °C	[59]
*spvB*	5′-CTATCAGCCCGGCACGGAGAGCAGTTTTTA-3′ 5′-GGAGGAGGCGGTGGCCGTGGCATCATA-3′	717	60 °C	[59]
*spiA*	5′-CCAGGGCTCGTTAGTGTATTGCGTGAGATG-3′ 5′-CGCGTAACAAAGAACCGGTAGTGATGGATT-3′	550	54 °C	[59]
*pagC*	5′-CGCCTTTTCCCTGGGGTATGC-3′ 5′-GAAGCGGTTTATTTTTGTAGAGGAGATGTT-3′	454	54 °C	[59]
*cdtB*	5′-ACAACTGTCGGATCTCGCCCCGTCATT-3′ 5′-CAATTTGCGTGGCTTCTGTAGGTGCGAGT-3′	268	54 °C	[59]
*sifA*	5′-TTTGCCGAACGCGGCCCCACACG-3′ 5′-GTTGCCTTTTCTTGCCCTTTCCACCCATCT-3′	440	65 °C	[59]
*sopB*	5′-CGGACCGGCCAGCAACCAAACAAGAAGAAG-3′ 5′-TAGTGATGCCCGTTATCCGTGAGTGTATT-3′	220	54 °C	[59]
*pefA*	5′-GCGCCGCTCAGCCGGACCAG-3′ 5′-GCAGCAGAAGCCCAGCAAACAGTG-3′	157	55 °C	[59]
*prgH*	5′-GCCCGAGCAGGCTGAGAAGTTAGAAA-3′ 5′-TGAAATGAGCGGCCCTTGAGCCAGTC-3′	756	55 °C	[59]
*SpaN*	5′-AAAAGCCCTGGAATCCGTTAGTGAAGT-3′ 5′-CAGCGCTGGGCATTACCGTTTTG-3′	504	60 °C	[59]
*orgA*	5′-TTTTTGGCCATGCATCAGGGAACA-3′ 5′-GGCGAAAGCGGGCACGGTATT-3′	255	55 °C	[59]

## References

[1] Fung F., Wang H.S., Menon S. (2018). Food safety in the 21st century. Biomed. J..

[2] Käferstein F. (2003). Foodborne diseases in developing countries: Aetiology, epidemiology and strategies for prevention. Int. J. Environ. Health Res..

[3] Bosilevac J.M., Guerini M.N., Kalchayanand N., Koohmaraie M. (2009). Prevalence and characterization of *Salmonella* in commercial ground beef in the United States. Appl. Environ. Microbiol..

[4] EFSA (2014). The European Union summary report on trends and sources of zoonoses, zoonotic agents and food-borne outbreaks in 2014. Efsa J..

[5] Mahmood A.K., Khan M.S., Khan M.A., Khan M.A., Bilal M. (2014). Prevalence of *Salmonella* in diarrheic adult goats in field conditions. J. Anim. Plant Sci..

[6] Antunes P., Mourão J., Campos J., Peixe L. (2016). Salmonellosis: The role of poultry meat. Clin. Microbiol. Infect..

[7] Balasubramanian R., Im J., Lee J.S., Jeon H.J., Mogeni O.D., Kim J.H., Marks F. (2019). The global burden and epidemiology of invasive non-typhoidal *Salmonella* infections. Hum. Vaccines Immunother..

[8] Obeid R., Heil S.G., Verhoeven M.M., Van Den Heuvel E.G., De Groot L.C., Eussen S.J. (2019). Vitamin B12 intake from animal foods, biomarkers, and health aspects. Front. Nutr..

[9] Carrie D., Cross A., Koebnick C., Sinha R. (2011). Trends in meat consumption in the United States. Public Health Nutr..

[10] Sohaib M., Jamil F. (2017). An insight of meat industry in Pakistan with special reference to halal meat: A comprehensive review. Korean J. Food Sci. Anim. Resour..

[11] Guerin M.T., Martin S.W., Darlington G.A., Rajic A. (2005). A temporal study of *Salmonella* serovars in animals in Alberta between 1990 and 2001. Can. J. Vet. Res..

[12] Gutema F.D., Agga G.E., Abdi R.D., Duchateau L., DeZutter L., Gabriël S. (2019). Prevalence and Serotype Diversity of *Salmonella* in apparently healthy Cattle: Systematic Review and Meta-Analysis of Published Studies, 2000–2017. Front. Vet. Sci..

[13] McDonough P.L., Fogelman D., Shin S.J., Brunner M.A., Lein D.H. (1999). *Salmonella enterica* serotype Dublin infection: An emerging infectious disease for the Northeaster United States. J. Clin. Microbiol..

[14] Molla B., Alemayehu D., Salah W. (2003). Sources and distribution of *Salmonella* serotypes isolated from food animals, slaughterhouse personnel and retail meat products in Ethiopia: 1997–2002. Ethiop. J. Health Dev..

[15] Garedew L., Hagos Z., Addis Z., Tesfaye R., Zegeye B. (2015). Prevalence and antimicrobial susceptibility patterns of *Salmonella* isolates in association with hygienic status from butcher shops in Gondar town, Ethiopia. Antimicrob. Resist. Infect. Control.

[16] Kamboh A.A., Shoaib M., Abro S.H., Khan M.A., Malhi K.K., Yu S. (2018). Antimicrobial Resistance in *Enterobacteriaceae* Isolated from Liver of Commercial Broilers and Backyard Chickens. J. Appl. Poult. Res..

[17] Monte D.F., Lincopan N., Fedorka-Cray P.J., Landgraf M. (2019). Current insights on high priority antibiotic-resistant *Salmonella enterica* in food and foodstuffs: A review. Curr. Opin. Food Sci..

[18] Cuypers W.L., Jacobs J., Wong V., Klemm E.J., Deborggraeve S., Van Puyvelde S. (2018). Fluoroquinolone resistance in *Salmonella*: Insights by whole-genome sequencing. Microb. Genom..

[19] Fernández J., Guerra B., Rodicio M.R. (2018). Resistance to carbapenems in non-typhoidal *Salmonella enterica* Serovars from humans, animals and food. Vet. Sci..

[20] MacDonald E., White R., Mexia R., Bruun T., Kapperud G., Brandal L.T., Vold L. (2019). The role of domestic reservoirs in domestically acquired *Salmonella* infections in Norway: Epidemiology of salmonellosis, 2000–2015, and results of a national prospective case–control study, 2010–2012. Epidemiol. Infect..

[21] Rotimi V.O., Jamal W., Pal T., Sonnevend A., Dimitrov T.S., Albert M.J. (2008). Emergence of multidrug-resistant *Salmonella* spp. and isolates with reduced susceptibility to ciprofloxacin in Kuwait and the United Arab Emirates. Diagn. Microbiol. Infect. Dis..

[22] Kariuki S., Gordon M.A., Feasey N., Parry C.M. (2015). Antimicrobial resistance and management of invasive *Salmonella* disease. Vaccine.

[23] Munir S., Ali S., Ali S. (2019). A systematic review on shifting trends of foodborne diseases in Pakistan. Abasyn J. Life Sci..

[24] Van T.T.H., Moutafis G., Istivan T., Tran L.T., Coloe P.J. (2007). Detection of *Salmonella* spp. in retail raw food samples from Vietnam and characterization of their antibiotic resistance. Appl. Environ. Microbiol..

[25] Soomro A.H., Khaskheli M., Bhutto M.B., Shah G., Memon A., Dewani P. (2011). Prevalence and antimicrobial resistance of *Salmonella* serovars isolated from poultry meat in Hyderabad, Pakistan. Turk. J. Vet. Anim. Sci..

[26] Samad A., Abbas F., Tanveer Z.U., Ahmad Z.A., Raziq A.B., Zahid M. (2018). Prevalence of Salmonella spp. in chicken meat from Quetta retail outlets and typing through multiplex PCR. Rom. Biotechnol. Lett..

[27] Almeida C., Cerqueira L., Azevedo N.F., Vieira M.J. (2013). Detection of *Salmonella enterica* serovar Enteritidis using real time PCR, immunocapture assay, PNA FISH and standard culture methods in different types of food samples. Int. J. Food Microbiol..

[28] Siala M., Barbana A., Smaoui S., Hachicha S., Marouane C., Kammoun S., Messadi-Akrout F. (2017). Screening and detecting *Salmonella* in different food matrices in southern Tunisia using a combined enrichment/real-time PCR method: Correlation with conventional culture method. Front. Microbiol..

[29] Barkocy-Gallagher G.A., Arthur T.M., Rivera-Betancourt M., Nou X., Shackelford S.D., Wheeler T.L., Koohmaraie M. (2003). Seasonal prevalence of Shiga toxin–producing *Escherichia coli*, including O157: H7 and non-O157 serotypes, and *Salmonella* in commercial beef processing plants. J. Food Prot..

[30] Sallam K.I., Mohammed M.A., Hassan M.A., Tamura T. (2014). Prevalence, molecular identification and antimicrobial resistance profile of *Salmonella* serovars isolated from retail beef products in Mansoura, Egypt. Food Control.

[31] El-Sharkaway M.S., Samaha I.A., Abd El Galil H.I. (2016). Prevalence of Pathogenic Microorganisms in Raw Meat Products from Retail Outlets in Alexandria Province. AJVS.

[32] Dallal M.M.S., Yazdi M.S., Mirzaei N., Kalantar E. (2014). Prevalence of *Salmonella* spp. in packed and unpacked red meat and chicken in south of Tehran. Jundishapur J. Microbiol..

[33] Hyeon J.Y., Chon J.W., Hwang I.G., Kwak H.S., Kim M.S., Kim S.K., Seo K.H. (2011). Prevalence, antibiotic resistance, and molecular characterization of *Salmonella* serovars in retail meat products. J. Food Prot..

[34] Jalali M., Abedi D., Pourbakhsh S.A., Ghoukasin K. (2008). Prevalence of *Ssalmonella* spp. in raw and cooked foods in Isfahan-Iran. J. Food Saf..

[35] Abouzeed Y.M., Hariharan H., Poppe C., Kibenge F.S. (2000). Characterization of *Salmonella* isolates from beef cattle, broiler chickens and human sources on Prince Edward Island. Comp. Immunol. Microbiol. Infect. Dis..

[36] Tafida S.Y., Kabir J., Kwaga J.K.P., Bello M., Umoh V.J., Yakubu S.E., Hendriksen R. (2013). Occurrence of *Salmonella* in retail beef and related meat products in Zaria, Nigeria. Food Control.

[37] Thai T.H., Yamaguchi R. (2012). Molecular characterization of antibiotic-resistant *Salmonella* isolates from retail meat from markets in Northern Vietnam. J. Food Prot..

[38] Thai T.H., Hirai T., Lan N.T., Shimada A., PHAM T.N., Yamaguchi R. (2012). Antimicrobial resistance of *Salmonella* serovars isolated from beef at retail markets in the north Vietnam. J. Vet. Med. Sci..

[39] Ur Rahman S., Mohsin M. (2019). The under reported issue of antibiotic-resistance in food-producing animals in Pakistan. Pak. Vet. J..

[40] Ekli R., Adzitey F., Huda N. (2019). Prevalence of resistant *Salmonella* spp. isolated from raw meat and liver of cattle in the Wa Municipality of Ghana. IOP Conference Series: Earth and Environmental Science.

[41] Maharjan M., Joshi V., Joshi D.D., Manandhar P. (2006). Prevalence of *Salmonella* species in various raw meat samples of a local market in Kathmandu. Ann. N. Y. Acad. Sci..

[42] Nhung N.T., Van N.T.B., Van Cuong N., Duong T.T.Q., Nhat T.T., Hang T.T.T., Campbell J. (2018). Antimicrobial residues and resistance against critically important antimicrobials in non-typhoidal *Salmonella* from meat sold at wet markets and supermarkets in Vietnam. Int. J. Food Microbiol..

[43] Yang X., Wu Q. (2019). Prevalence, bacterial load, and antimicrobial resistance of *Salmonella* serovars isolated from retail meat and meat products in China. Front. Microbiol..

[44] Kebede A., Kemal J., Alemayehu H., Habte Mariam S. (2016). Isolation, identification, and antibiotic susceptibility testing of *Salmonella* from slaughtered bovines and ovines in Addis Ababa Abattoir Enterprise, Ethiopia: A cross-sectional study. Int. J. Bacteriol..

[45] McEvoy J.M., Doherty A.M., Finnerty M., Sheridan J.J., McGuire L., Blair I.S., Harrington D. (2000). The relationship between hide cleanliness and bacterial numbers on beef carcasses at a commercial abattoir. Lett. Appl. Microbiol..

[46] Reid C.A., Small A., Avery S.M., Buncic S. (2002). Presence of food-borne pathogens on cattle hides. Food Control.

[47] Zhang L., Fu Y., Xiong Z., Ma Y., Wei Y., Qu X., Liao M. (2018). Highly prevalent multidrug-resistant *Salmonella* from chicken and pork meat at retail markets in Guangdong, China. Front. Microbiol..

[48] D’Aoust J.Y., Doyle M.P., Beuchat L.R., Montville T.J. (1997). *Salmonella* pecies. Food Microbiology Fundamentals and Frontiers.

[49] Li R., Lai J., Wang Y., Liu S., Li Y., Liu K., Wu C. (2013). Prevalence and characterization of *Salmonella* species isolated from pigs, ducks and chickens in Sichuan Province, China. Int. J. Food Microbiol..

[50] Yanestria S.M., Rahmaniar R.P., Wibisono F.J., Effendi M.H. (2019). Detection of *invA* gene of *Salmonella* from milkfish (*Chanos chanos*) at Sidoarjo wet fish market, Indonesia, using polymerase chain reaction technique. Vet. World.

[51] Rahn K., De Grandis S.A., Clarke R.C., McEwen S.A., Galan J.E., Ginocchio C., Gyles C.L. (1992). Amplification of an *invA* gene sequence of *Salmonella* Typhimurium by polymerase chain reaction as a specific method of detection of *Salmonella*. Mol. Cell. Probes.

[52] Raffatellu M., Wilson R.P., Chessa D., Andrews-Polymenis H., Tran Q.T., Lawhon S., Bäumler A.J. (2005). *SipA*, *SopA*, *SopB*, *SopD*, and *SopE2* contribute to *Salmonella enterica* serotype Typhimurium invasion of epithelial cells. Infect. Immun..

[53] Wajid M., Awan A.B., Saleemi M.K., Weinreich J., Schierack P., Sarwar Y., Ali A. (2019). Multiple drug resistance and virulence profiling of *Salmonella enterica* serovars Typhimurium and Enteritidis from poultry farms of Faisalabad, Pakistan. Microb. Drug Resist..

[54] Seyedjavadi S.S., Goudarzi M., Sabzehali F. (2016). Relation between *blaTEM*, *blaSHV* and *blaCTX*-M genes and acute urinary tract infections. J. Acute Dis..

[55] Van Boeckel T.P., Glennon E.E., Chen D., Gilbert M., Robinson T.P., Grenfell B.T., Laxminarayan R. (2017). Reducing antimicrobial use in food animals. Science.

[56] Kiiru J., Kariuki S., Goddeeris B.M., Butaye P. (2006). Analysis of β-lactamase phenotypes and carriage of selected β-lactamase genes among *Escherichia coli* strains obtained from Kenyan patients during an 18-year period. BMC Microbiol..

[57] Miró E., Navarro F., Mirelis B., Sabaté M., Rivera A., Coll P., Prats G. (2002). Prevalence of clinical isolates of *Escherichia coli* producing inhibitor-resistant β-lactamases at a University Hospital in Barcelona, Spain, over a 3-year period. Antimicrob. Agents Chemother..

[58] Warjri I., Dutta T.K., Lalzampuia H., Chandra R. (2015). Detection and characterization of extended-spectrum β-lactamases (*blaCTX*-*M-1* and *blaSHV*) producing *Escherichia coli*, *Salmonella* spp. and *Klebsiella pneumoniae* isolated from humans in Mizoram. Vet. World.

[59] Skyberg J.A., Logue C.M., Nolan L.K. (2006). Virulence genotyping of *Salmonella* spp. with multiplex PCR. Avian Dis..

